# Mutualisms as a framework for multi-robot collaboration

**DOI:** 10.3389/frobt.2025.1566452

**Published:** 2025-03-28

**Authors:** Alexander A. Nguyen, Mauriel Rodriguez Curras, Magnus Egerstedt, Jonathan N. Pauli

**Affiliations:** ^1^ Samueli School of Engineering, University of California Irvine, Irvine, CA, United States; ^2^ Department of Forest and Wildlife Ecology, University of Wisconsin-Madison, Madison, WI, United States

**Keywords:** mutualisms, multi-robot systems, heterogeneity, landscape, fitness

## Abstract

Biology has inspired robotics since its inception as an academic discipline. However, the use of ecological principles in robotics is still relatively rare and in this paper, we explore how such principles can not only be of relevance to robotics, but can reciprocally lead to new insights into ecology. In particular, we investigate how mutualisms–jointly beneficial interactions between members of different species–can inform collaborative architectures for multi-robot systems comprised of different types of robots. To better understand how mutualisms can have practical relevance in robotics, we present a case study where the landscape heterogeneity, i.e., the configuration of the landscape, is varied, and we measure the efficiency of robots functioning independently or involved in a mutualism. We show that landscape composition impacts the benefits of forming mutualisms, which, in turn, has implications for mutualism emergence and stability in ecology. Moreover, through this case study, the concept of fitness and its components can be introduced for engineered systems, leading to notions of longevity, task fecundity, and, ultimately, robot fitness.

## 1 Introduction

Collaboration is foundational to functioning societies and has been studied in diverse settings and disciplines, such as collective behaviors among eusocial insects or human team formation (Wilson, 1971; Camazine et al., 2001; Bowles and Gintis, 2011). In robotics, some tasks are more achievable when multiple robots are deployed; e.g., a disaster area can more effectively be searched and secured if more than a single robot is involved, and multiple robots can manipulate larger and heavier loads together. Additionally, if the robot team is heterogeneous, tasks can be accomplished by working together that would otherwise not be possible by any individual robot alone (Tuci et al., 2018). These observations point towards the importance of a formal understanding of the efficacy of multi-robot collaboration.

In ecology, collaborations are conceptualized as mutualisms–jointly beneficial interactions between members of different species (Pauli et al., 2014). Mutualisms are emergent properties in that the species involved can gain novel capabilities without evolving the traits themselves (Bronstein, 2009). Minimally, the benefits gained must be greater than the associated costs for mutualisms to emerge and, subsequently, persist (Leigh Jr, 2010). In addition, mutualisms enhance biodiversity by providing novel evolutionary trajectories and by increasing the available biomass and energy of ecological communities. Canonical examples of mutualisms include arbuscular mycorrhizal fungi and plants (Kiers and Heijden, 2006), figs (*Ficus* spp.) and fig wasps (family Agaonidae) (Cook and Rasplus, 2003), and whistling-thorn trees (*Vachellia drepanolobium*) and native acacia ants (*Crematogaster* spp.) (Kamaru et al., 2024).

Extending G. Evelyn Hutchinson’s classic metaphor of the ecological theater (Hutchinson, 1965) but featuring different players (organisms and robots) and stage (landscape and workspace), the *play* (interactions and interplay between individuals) is nevertheless the same in ecology and robotics. To that end, if robotics can benefit from an understanding of when collaborations are beneficial–a well-established principle in ecology–two questions arise: “How can collaboration (i.e., mutualisms), viewed from an ecological vantage point, be leveraged in robotics settings?” and “How can an understanding of collaborations in robotics contribute to ecology?”. Starting with mutualisms as a framework, this paper shows that its transition from ecology to robotics is not only relevant to heterogeneous multi-robot systems, but can also lead to other concepts not previously explored in robotics, such as robot fitness and its underlying components of longevity and task fecundity. Reciprocally, we show that ecology can, in turn, benefit from the findings of experiments in robotics to elucidate how landscape composition can affect the stability of mutualisms.

## 2 Collaboration in ecology and robotics

### 2.1 Ecological mutualisms

Mutualisms define many ecosystem processes. The products and services being exchanged, however, can vary widely, from nutrients and shelter to protection and pollination (Cushman and Beattie, 1991). These mutually beneficial arrangements emerge due to evolutionary pressures, where the participating partners gain an advantage through their association, with the three primary mechanisms of interdependent interactions: partner fidelity, feedback loops, and partner choice (Bronstein, 2009). Such mutualisms can take on various forms, ranging from obligate, where partners rely exclusively on each other, to facultative, where partners can survive without relying on each other (Chomicki et al., 2020).

Mutualism stability occurs when the benefits outweigh the costs for both participants, fostering sustained collaboration (Chomicki et al., 2020). However, disruptions to environmental conditions, the availability of resources, or the behavior of one partner (cheating) can lead to asymmetries, causing the mutualism to break down (Sachs and Simms, 2006). For example, the recent invasion of a novel agent, the big-headed ant (*Pheidole megacephala*), to a savanna ecosystem disrupted the mutualism between native acacia ants (*C.* spp.) and the whistling-thorn trees (*V. drepanolobium*). Without defense, the trees became susceptible to elephant (*Loxodonta africana*) browsing, which resulted in tree suppression and the opening of landscapes featuring high visibility and a subsequent shift in the predator-prey dynamics between lions (*Panthera leo*) and zebra (*Equus quagga*) (Kamaru et al., 2024). This example illustrates how mutualisms can dramatically reshape the landscape with cascading and ecosystem-wide consequences.

Resource gradients, abiotic (stress) gradients, and the distribution of mutualists across a system have been theoretically and empirically shown to influence mutualism stability and the forms that mutualisms take (Hoeksema and Bruna, 2015; Rogalski et al., 2021; Hauert and Doebeli, 2004). However, a largely open question in ecology is how landscape heterogeneity (i.e., the configuration of the landscape independent of resource and stress quantities) influences mutualism occurrence and persistence.

### 2.2 Multi-robot coordination

Multi-robot coordination concerns itself with how to make teams of robots achieve a common objective through the design of appropriate, individual strategies (Yan et al., 2013). A distinction, however, is typically made between homogeneous and heterogeneous teams (Verma and Ranga, 2021). In robotics, heterogeneity is commonly understood along the following dimensions (Notomista et al., 2021): mobility (how is the robot moving through the environment), sensing (what means does the robot possess to gather information about its environment), computation (how effectively can the obtained information be processed), and communication (how do the robots share information between each other).

It has been observed that heterogeneous robot teams are better suited to tackle more complex problems than their homogeneous counterparts (Parker, 1994; Jones et al., 2006; Mayya et al., 2021). The traditional approach to heterogeneous task assignment decomposes the mission into subtasks that can be solved by the individual robots based on their respective capabilities (Rizk et al., 2019). However, such an approach fails to expand the set of capabilities that collaboration might enable, and the whole is not greater than the sum of its parts. Or, to borrow terminology from ecology, the robots cannot gain novel capabilities without “evolving” the corresponding traits themselves. To that end, we focus on collaboration in heterogeneous teams rather than on the relatively better-studied question of how to coordinate activities among homogeneous robots. For collaborative arrangements to be preferred, there must be a clear motivation for why robots should work together in the first place. In this paper, we will examine how the environment can provide insights into when collaborations would be beneficial, reminiscent of the role that the landscape plays in ecology (Pianka, 1994).

### 2.3 Transferability across disciplines

Fitness is a universal concept and is relevant to many disciplines. In evolutionary ecology, fitness is a function of an individual’s survival and reproductive output. Although it is a population parameter, fitness can still be estimated for individuals who can boost their overall fitness by increasing their survival, i.e., lifespan, or by improving their reproductive output, i.e., number of offspring (Doak et al., 2002).

Unlike biological systems that emerge through natural selection over millennia, engineered systems are designed with a particular purpose in mind–scissors are built to cut, while transistors are meant to act as switches in integrated circuits. However, the central concept of fitness still applies. We propose that robot fitness, based on the similar ecological underpinnings of organismal fitness, is a useful framework for multi-robot systems (Egerstedt et al., 2018; Egerstedt, 2021). Within this framework, we conceptualize robot fitness as a function of two components: longevity, i.e., the duration over which a robot can perform its tasks, and task fecundity, i.e., the rate at which tasks can be completed. These two characteristics–longevity (akin to survival) and task fecundity (akin to reproduction) – enable a construct by which the disparate disciplines of robotics and ecology can share a common language through which the value of collaboration can be formulated and understood.

## 3 Case study: A test of landscape heterogeneity in robotics

### 3.1 Traversing through different landscape profiles

To elucidate how mutualisms can have practical relevance in robotics, we consider a case study where the landscape heterogeneity shapes the collaborations among the robots while, at the same time, providing feedback to ecology to better understand how mutualisms may arise. We consider an idealized experiment in which two robots with different mobility characteristics are tasked with reaching goal points either independently or collaboratively (i.e., involved in a mutualism). When the robots act independently, they simply move towards their respective goal points while collaboration involves one robot carrying the other. One would expect that if the robots form mutualisms, they might complete their respective tasks utilizing less energy and, therefore, have greater longevity and exhibit higher task fecundity. However, as will be seen, the degree of landscape heterogeneity and the cost associated with forming the mutualism can negate these potential benefits.

**FIGURE 1:**
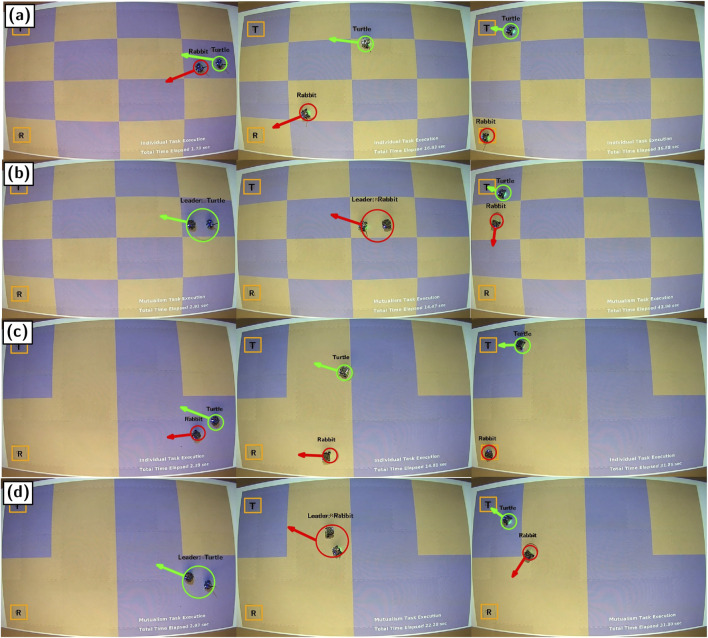
Snapshots from the conducted experimental case study. One robot is more effective on land (the “rabbit”; $R_{\text{rabbit}}$ ) while another is more effective in water (the “turtle”; $R_{\text{turtle}}$ ). These robots either work independently or collaboratively (i.e., involved in a mutualism) to reach their respective goal points (orange squares with ‘R’ for $R_{\text{rabbit}}$ and ‘T’ for $R_{\text{turtle}}$ ) within either high or low-variability landscapes. In **(a)**, $R_{\text{rabbit}}$ and $R_{\text{turtle}}$ work independently to reach their respective goal points within a high-variability landscape. In **(b)**, $R_{\text{rabbit}}$ and $R_{\text{turtle}}$ form a mutualism to reach their respective goal points within a high-variability landscape. In **(c)**, $R_{\text{rabbit}}$ and $R_{\text{turtle}}$ work independently within a low-variability landscape, and in **(d)**, they form a mutualism within a low-variability landscape.

The case study is designed as a sequence of empirical robotics experiments, where mobile robots operate in a workspace with features that can be modified as needed. In particular, we consider the scenario where the environment is artificially discretized into a finite number of pixels of two different types, representing resistance, distributed into high-variability and low-variability patterns across four different landscape profiles. For illustrative purposes, we refer to these two terrain types as “water” and “land” (depicted as blue and brown pixels, respectively, in Figure 1), where all the pixels of each type are gathered into the sets $D_{\text{water}}$ and $D_{\text{land}}$ . High-variability patterns exhibit landscape profiles where the terrain types alternate frequently (as portrayed in Figures 1A,B), while low-variability patterns exhibit landscape profiles where terrain types alternate infrequently (as portrayed in Figures 1C,D).

The premise behind the experiments is that they should clearly point out the potential benefits (or lack thereof) associated with collaborative arrangements. To that end, we assume that both robots can traverse either terrain type, but they incur different energetic costs in that one robot is more effective on land while the other is more effective in water. Continuing the water and land analogy, we call the robot with a land preference the “rabbit” $\left(R_{\text{rabbit}}\right)$ , while the other robot is referred to as the “turtle” $\left(R_{\text{turtle}}\right)$ . Not only is it energetically beneficial for $R_{\text{rabbit}}$ to reside in $D_{\text{land}}$ , it can also move faster in that domain than in $D_{\text{water}}$ , while the opposite holds for $R_{\text{turtle}}$ .

The mutualism comes into play by allowing the robots to carry each other. By doing so, the robot performing the carrying incurs an additional energetic cost, while the movement becomes energetically free for the carried robot. Additionally, the robots’ speeds are also affected by the mutualism. The resulting speeds are given in descending order for $R_{\text{rabbit}}$ by

$$v_{\text{land}}^{\text{rabbit}\text{ alone}}>v_{\text{land}}^{\text{rabbit}\text{ carries }\text{turtle}}>v_{\text{water}}^{\text{rabbit}\text{ alone}}>v_{\text{water}}^{\text{rabbit}\text{ carries }\text{turtle}}$$ (1)

This means that $R_{\text{rabbit}}$ is at its fastest when moving alone on land. Its slowest modality occurs when it is carrying the turtle in the water. Similarly, the speeds for $R_{\text{turtle}}$ are given by

$$v_{\text{water}}^{\text{turtle}\text{ alone}}>v_{\text{water}}^{\text{turtle}\text{ carries }\text{rabbit}}>v_{\text{land}}^{\text{turtle}\text{ alone}}>v_{\text{land}}^{\text{turtle}\text{ carries }\text{rabbit}}$$ (2)

Rather than having the robots physically carry each other, “carrying” is represented in the experiments by the robots being in close proximity, organized in such a way that one robot leads the other. In the collaborative scenario, the decision of which robot assumes the role of the leader is dependent on the terrain in that energetically beneficial arrangements are always preferred. Namely, when in a collaborative arrangement, $R_{\text{rabbit}}$ carries $R_{\text{turtle}}$ in $D_{\text{land}}$ , while $R_{\text{turtle}}$ carries $R_{\text{rabbit}}$ in $D_{\text{water}}$ . Each such collaborative arrangement is classified as a “mode” and, in addition to the situation in which $R_{\text{rabbit}}$ carries $R_{\text{turtle}}$ (and visa versa), there are also modes associated with setting up or separating after the collaboration concludes.

### 3.2 Energetic expenditure parameterization

To evaluate the effects of collaboration on how well the robots are traversing through the environment, additional parameters are needed (Table 1). These are the robot mass (M) , the landscape pixel resistance (c) , the energetic cost $\left(\delta_{\text{switch}}\right)$ associated with setting up or disengaging from the collaborative mode, and the initial energy stored in each robot’s battery when fully charged $\left(E_{0}\right)$ . Although the particular values associated with these parameters do not matter significantly to the outcomes of the experiments, they are chosen to be physically realistic. As the energetic cost associated with a particular path has to be evaluated along its entirety, the movement cost for $R_{\text{rabbit}}$ and $R_{\text{turtle}}$ are given by the path integrals.

$$E_{\text{rabbit}}=\int_{P_{\text{rabbit}}}m_{\text{rabbit}}\left(p\right)c_{\text{rabbit}}\left(p\right)dp,$$ (3)

$$E_{\text{turtle}}=\int_{P_{\text{turtle}}}m_{\text{turtle}}\left(p\right)c_{\text{turtle}}\left(p\right)dp,$$ (4)

where $P_{\text{rabbit}}$ and $P_{\text{turtle}}$ are the paths taken by the robots in order to reach their respective goal points. Here, the pointwise masses are given by.

$$m_{\text{rabbit}}\left(p\right)=\left\{\begin{array}{cc} M_{\text{rabbit}}+M_{\text{turtle}}\; & \text{if }\;R_{\text{rabbit}}\;\text{ carries }\;R_{\text{turtle}}\;\text{ at}\;\;p\\ M_{\text{rabbit}}\; & \text{if }\;R_{\text{rabbit}}\;\text{moves}\;\text{alone}\;\;\text{at }\;\;p\\ 0\; & \text{if }\;R_{\text{turtle}}\;\text{ carries }\;R_{\text{rabbit}}\;\text{ at}\;p \end{array}\right.$$ (5)

$$m_{\text{turtle}}\left(p\right)=\left\{\begin{array}{cc} M_{\text{turtle}}+M_{\text{rabbit}}\; & \;\text{if }R_{\text{turtle}}\;\text{ carries }\;R_{\text{rabbit}}\;\text{ at }\;p\\ M_{\text{turtle}}\; & \text{if }\;R_{\text{turtle}}\;\text{ moves alone at }\;p\\ 0\; & \text{if }\;R_{\text{rabbit}}\;\text{ carries }\;R_{\text{turtle}}\;\text{ at}\;p \end{array}\right.$$ (6)

In addition, the pointwise pixel resistances are given by.

$$c_{\text{rabbit}}\left(p\right)=\left\{\begin{array}{cc} c_{\text{rabbit}}^{\text{water}}\; & \text{if }\;p\in D_{\text{water}}\\ c_{\text{rabbit}}^{\text{land}}\; & \text{if }\;p\in D_{\text{land}} \end{array}\right.$$ (7)

$$c_{\text{turtle}}\left(p\right)=\left\{\begin{array}{cc} c_{\text{turtle}}^{\text{water}}\; & \text{if }\;p\in D_{\text{water}}\\ c_{\text{turtle}}^{\text{land}}\; & \text{if }\;p\in D_{\text{land}} \end{array}\right.$$ (8)

where $c_{\text{rabbit}}^{\text{water}}$ and $c_{\text{turtle}}^{\text{water}}$ are the robots’ pixel resistances in $D_{\text{water}}$ , while $c_{\text{rabbit}}^{\text{land}}$ and $c_{\text{turtle}}^{\text{land}}$ are the robots’ pixel resistances in $D_{\text{land}}$ .

**TABLE 1:** Experimental settings.

Parameters	Values	Unit
$\left\{M_{\text{rabbit}},\;M_{\text{rabbit}}\right\}$	{0.265, 0.265}	kg
$\left\{v_{\text{land}}^{\text{rabbit}\text{ alone}},\;v_{\text{land}}^{\text{rabbit}\text{ carries }\text{turtle}}\right\}$	{19.2, 18.24}	cm/s
$\left\{v_{\text{water}}^{\text{rabbit}\text{ alone}},\;v_{\text{water}}^{\text{rabbit}\text{ carries }\text{turtle}}\right\}$	{8, 7.6}	cm/s
$\left\{v_{\text{water}}^{\text{turtle}\text{ alone}},\;v_{\text{water}}^{\text{turtle}\text{ carries }\text{rabbit}}\right\}$	{14.4, 13.68}	cm/s
$\left\{v_{\text{land}}^{\text{turtle}\text{ alone}},\;v_{\text{land}}^{\text{turtle}\text{ carries }\text{rabbit}}\right\}$	{6.4, 6.08}	cm/s
$\left\{c_{\text{rabbit}}^{\text{land}},\;c_{\text{turtle}}^{\text{land}}\right\}$	{1, 10}	J kg^-1^m^-1^
$\left\{c_{\text{rabbit}}^{\text{water}},\;c_{\text{turtle}}^{\text{water}}\right\}$	{10, 1}	J kg^-1^m^-1^
$\delta_{\text{switch}}$	0.95	J
$E_{0}$	37,800	J

In robotics, as in ecology, setting up and disengaging from a collaborative arrangement is not free. If $N_{\text{switch}}$ is the total number of mode transitions performed along $P_{\text{rabbit}}$ and $P_{\text{turtle}}$ , the energy expenditures associated with the mode transitions becomes

$$E_{\text{switch}}=\delta_{\text{switch}}N_{\text{switch}}$$ (9)

For simplicity, we assume that this cost is experienced the same by both robots. In fact, it should be pointed out that unlike the other parameters in the experiment, the cost to form a mutualism is not directly coupled to any physical parameters such as mass, distance, or pixel resistance, meaning that this value acts as a scale factor that does not qualitatively affect the outcome of the experiments.

The total energy expenditure of each robot is now given as the sum of energy expended while moving ( $E_{\text{rabbit}}$ or $E_{\text{turtle}}$ ) and during mode transitions $\left(E_{\text{switch}}\right)$ . Furthermore, energy expenditure is directly linked to battery life, i.e., how long a robot can keep performing tasks. Thus, we explicitly link longevity, L , to energy expenditures in the sense that.

$$L_{\text{rabbit}}=\frac{E_{0}}{E_{\text{rabbit}}+E_{\text{switch}}},$$ (10)

$$L_{\text{turtle}}=\frac{E_{0}}{E_{\text{turtle}}+E_{\text{switch}}},$$ (11)

where $E_{0}$ is the initial energy capacity of the robots’ batteries, and where we assume a uniform battery discharge model for the robots (Morris and Tosunoglu, 2012).

Longevity by itself does not tell the full story. It is entirely possible that a robot can move around for a very long period of time without achieving any goal points, i.e., achieving any tasks. As such, the rate at which tasks are achieved is an important measure of the overall ability of the individual robots and, borrowing from the ecological concept of fecundity, we can use task fecundity, T , to emphasize this connection to ecology. In the context of the case study, task fecundity translates to the rate at which tasks (i.e., goal points) are achieved, given by tasks completed per second, as.

$$T_{\text{rabbit}}=\frac{\rho_{\text{rabbit}}}{t_{\text{rabbit}}}$$ (12)

$$T_{\text{turtle}}=\frac{\rho_{\text{turtle}}}{t_{\text{turtle}}}$$ (13)

Here, $\rho_{\text{rabbit}}$ and $\rho_{\text{turtle}}$ are the total number of goal points that are achieved by the respective robots throughout a deployment, while $t_{\text{rabbit}}$ and $t_{\text{turtle}}$ are the times it took them to reach these goal points.

We can now describe how long a robot can operate (longevity) and the rate at which tasks are being solved (task fecundity). The final, missing piece is the total number of tasks solved, i.e., a notion closely related to fitness. To that end, we define robot fitness, F , as longevity times task fecundity,

$$F_{\text{rabbit}}=L_{\text{rabbit}}T_{\text{rabbit}},$$ (14)

$$F_{\text{turtle}}=L_{\text{turtle}}T_{\text{turtle}}.$$ (15)

With these entities established, we can now proceed to investigate how efficiently the robots move across the landscape and what the effects of collaborating (i.e., forming a mutualism) are within high-variability and low-variability environments.

### 3.3 Findings

**FIGURE 2:**
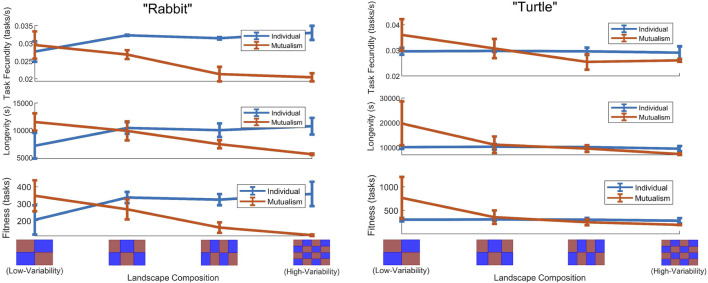
Task fecundity, longevity, and fitness quantities (on average) with error bars (±1σ) when the landscape composition (i.e., distribution pattern of pixels) varies across four different landscape profiles for a robot more effective on land (the “rabbit”; $R_{\text{rabbit}}$ ) and a robot more effective in water (the “turtle”; $R_{\text{turtle}}$ ) that functions either independently or collaboratively (i.e., involved in a mutualism).

By executing multiple experiments for each of the four different landscape profiles, as a way of describing the spectrum of variability, we found that (on average) the task fecundity, longevity, and fitness of both robots were higher when they engaged in a mutualism within low-variability landscapes while this did not hold in high-variability landscapes. Specifically, as the landscape became less varied, task fecundity, longevity, and fitness decreased for $R_{\text{rabbit}}$ when it was functioning independently, while all these quantities increased when collaborating with $R_{\text{turtle}}$ (Figure 2). In other words, it was highly beneficial for the rabbit to be carried by the turtle in the water, and this benefit was greater than the corresponding cost of carrying the turtle on land. However, as the number of transitions increases, the switching costs start to outweigh these benefits. At some point, the switching costs outweighed these benefits, making the individual approach preferable in high-variability environments. These benefits were also experienced by the turtle similarly (Figure 2). Note that the pixel arrangement–comprised of water (blue pixels) and land (brown pixels) – for each landscape profile is displayed on the horizontal axis (i.e., landscape composition; distribution pattern of pixels) of Figure 2.

Returning to the question of whether or not collaborations are beneficial in multi-robot systems, for this particular case study, the robots did exhibit a crossover point of benefit in task fecundity, longevity, and fitness for the individual and mutualism scenarios that was dependent on the landscape composition and total energy expenditures. Meaning as the landscape became less varied, collaborative arrangements became more beneficial. Note that the corresponding crossover point for $R_{\text{turtle}}$ occurred before that of $R_{\text{rabbit}}$ as the landscape became less varied, i.e., $R_{\text{turtle}}$ would find it beneficial to collaborate sooner than $R_{\text{rabbit}}$ would. These results show that, at least in this particular setting, the environment plays a significant role in deciding when it is beneficial for the robots to form a mutualism and when it is not.

## 4 “EcoBotics”

The experimental case study illustrates how ecological ideas can be employed to inform robotics. In particular, the concepts of fitness and its components lead to notions of longevity, task fecundity, and, ultimately, robot fitness (longevity times task fecundity). Under this framework, the emergence of mutualisms as a potentially beneficial strategy becomes apparent and highlights the landscape’s prominent role in promoting or impeding the formation of jointly beneficial associations. Furthermore, this approach also helps to uncover the particular fitness component that changes the most from a mutualism.

In conceptualizing robot fitness, the results suggest that roboticists can choose alternative strategies that lead to similar outcomes (MacArthur and Wilson, 1967; Pianka, 1970). There are conceivable scenarios in which the task is paramount, regardless of a robot’s longevity. Alternatively, there are cases where longevity and the continual presence of a robot would be prioritized, regardless of the number of tasks completed. The proposed collaborative framework thus provides support for roboticists to choose which of these are preferred or if overall fitness, i.e., the number of tasks completed during the lifetime of a robot, is what matters.

On the other hand, explaining mutualism emergence and stability in ecology has been challenging (Heath and Stinchcombe, 2014), and the impact of landscape configuration on mutualism emergence, performance, and stability has not been previously addressed. In fact, spatial heterogeneity between participants in the mutualism can lead to covarying effects between host and symbiont resource exploitation and, hence, reproductive success (Boza and Scheuring, 2004). Our robotics experiments showed that this observation can be extended to resource acquisition between potential mutualists within a heterogeneous environment independent of resource quantity. Specifically, the results from this idealized experiment reinforce the degree to which environmental contexts can shape mutualism emergence, performance, and stability.

Landscape heterogeneity has been shown to drive many facets of ecological interactions. Our results suggest that mutualism stability may be promoted (or minimally mediated) indirectly by environmental configuration, whereby higher costs are incurred by individuals who attempt to exploit the landscape independently while cooperating species utilize less energy to accomplish the same tasks. Notably, a similar concept in a heterogeneous resource environment created by figs has been previously observed, whereby fig hosts present wasp symbionts with a heterogeneous resource environment, which results in higher costs to exploitative individuals than the cooperative wasps that “allow” their host to set seeds (Yu et al., 2004). Broadly, our results suggest that mutualisms may have evolved in response to (or minimally been reinforced by) resource clumping, a well-studied feature of ecological systems (Wiens, 1989).

While cross-pollination between robotics and ecology is not new (Pauli and Egerstedt, 2021), this paper highlights that such collaborations can lead to new insights of relevance across disciplines. In particular, we found that this cross-disciplinary framework, termed “EcoBotics,” provides new insights into both fields. As demonstrated, the ecological concepts of mutualisms and fitness can provide novel insights into heterogeneous multi-robot systems. Accordingly, there are likely other applications of ecological concepts in robotics–such as an ecological niche or the metabolic theory of ecology–which, in turn, can be used to inform mechanisms driving ecological relationships.

## Data Availability

The datasets generated and analyzed for this study can be found in the following online repository: https://github.com/alexngxyen/From-Ecology-to-Robotics-and-Back-Mutualisms-as-a-Framework-for-Multi-Robot-Collaboration

## Supplementary material

SUPPLEMENTARY VIDEO S1 Hardware experiment in a high-variability landscape for robots functioning collaboratively.

SUPPLEMENTARY VIDEO S2 Hardware experiment in a low-variability landscape for robots functioning individually.

SUPPLEMENTARY VIDEO S3 Hardware experiment in a low-variability landscape for robots functioning collaboratively.

SUPPLEMENTARY VIDEO S4 Hardware experiment in a high-variability landscape for robots functioning individually.
